# Simultaneous Quantification of Brigatinib and Brigatinib-Analog in Rat Plasma and Brain Homogenate by LC-MS/MS: Application to Comparative Pharmacokinetic and Brain Distribution Studies

**DOI:** 10.1155/2019/9028309

**Published:** 2019-12-05

**Authors:** Bo Li, Min Lu, Lei Jin, Maoen Zheng, Peilu Sun, Shanshan Lei, Shan Xiong, Suhong Chen

**Affiliations:** ^1^Zhejiang University of Technology, Hangzhou 310014, China; ^2^School of Medicine and Life Sciences, University of Jinan-Shandong Academy of Medical Sciences, Jinan 250200, China; ^3^Department of Pharmaceutical Engineering, Shandong Drug and Food Vocational College, Weihai 264210, China; ^4^Shandong Laboratory Animal Center, Shandong First Medical University & Shandong Academy of Medical Sciences, Jinan 250002, China; ^5^Institute of Materia Medica, Shandong First Medical University & Shandong Academy of Medical Sciences, Jinan 250062, China

## Abstract

Brigatinib and brigatinib-analog are potent and selective ALK inhibitors with the similar structure. A simple and sensitive high-performance liquid chromatography with tandem mass spectrometry (LC-MS/MS) method for simultaneous determination of brigatinib and brigatinib-analog in rat plasma and brain homogenate was developed and validated. Chromatographic separation was carried out on an ODS column with acetonitrile and 0.1% formic acid in water as the mobile phase with gradient elution at a flow rate of 0.5 mL/min. Detections were performed using a TSQ Quantum Ultra mass spectrometric detector with electrospray ionization (ESI) interface, which was operated in the positive ion mode. A simple protein precipitation preparation process was used. The lower limits of quantification (LLOQs) were 1.0 ng/mL and 0.5 ng/mL for analytes in rat plasma and brain homogenate, respectively. The intrabatch and interbatch precision and accuracy of brigatinib and brigatinib-analog were well within the acceptable limits of variation. The simple and sensitive LC-MS/MS method was successfully applied to the pharmacokinetic and brain distribution studies following a single oral administration of brigatinib and brigatinib-analog to rats. The above studies would lay a good foundation for the further applications of brigatinib and brigatinib-analog.

## 1. Introduction

Lung cancer is one of the most commonly diagnosed tumors with high morbidity and mortality worldwide [1], and its incidence and mortality continue to grow. Lung cancer is divided into small-cell lung cancer (SCLC) and non-small-cell lung cancer (NSCLC), of which NSCLC accounts for 85% of lung cancer, including squamous cell carcinoma, adenocarcinoma, and large cell undifferentiated cancer [2, 3]. SCLC accounts for only 15%. Compared with SCLC, NSCLC has slower metastasis and proliferation. About 75% of NSCLC patients were found to be at the middle and late stages, and the 5-year reported survival rate was extremely low [4, 5].

The development of molecular research in the nearly ten years has meant significant breakthroughs in the diagnosis, detection, and treatment of lung cancer of the NSCLC. Anaplastic lymphoma kinase (ALK), a receptor tyrosine kinase of the insulin receptor family, was originally identified as a part of the fusion protein nucleophosmin-anaplastic lymphoma kinase (NPM-ALK). The gene rearrangement between ALK and echinoderm microtubule-associated protein-like 4 (EML4) becomes more general than ALK gene rearrangement, which is no more than 5% in advanced NSCLC. The constitutive kinase activity of the final product with carcinogenicity (EML4-ALK) represents the growth of ALK-rearranged (ALK-positive) NSCLC [6–8].

As one of the second-generation ALK inhibitors, brigatinib (AP26113), approved by FDA in April 2017, is a highly selective and efficient ALK inhibitor to treat patients with ALK-positive metastatic NSCLC and can overcome the acquired crizotinib resistance to the first-generation ALK inhibitor, especially the L1196M gatekeeper mutation [9, 10]. Brigatinib-analog (AP26113-analog, ALK-IN-1) is an orally active, potent and selective ALK, and the epidermal growth factor receptor (EGFR) inhibitor with the similar structure to brigatinib. Brigatinib-analog binds to and inhibits ALK kinase and ALK fusion proteins as well as EGFR and mutant forms. This leads to the inhibition of ALK kinase and EGFR kinase, disrupts their signaling pathways, and eventually inhibits tumor cell growth in susceptible tumor cells [3, 11].

It was reported that brigatinib and brigatinib-analog had similar potency against the triple mutation with IC50 values of <100 nM. In addition, the brigatinib and brigatinib-analog play a therapeutic role for brain metastases due to ability to reach the central nervous system (CNS) through the blood-brain barrier [12, 13].

To the best of our knowledge, there were only a few studies reported to determine the concentration of brigatinib in plasma and tissues [14, 15], and this was the first research reported for simultaneous determination of brigatinib and brigatinib-analog in rat plasma and brain homogenate using an LC-MS/MS method. As the primary purpose of the study, a sensitive, reliable, and simple LC-MS/MS method was established and validated for the simultaneous quantification of brigatinib and brigatinib-analog in rat plasma and brain homogenate. Also, the method was successfully applied to the pharmacokinetic and brain distribution studies of brigatinib and brigatinib-analog following a single oral administration to rats. This research would provide the foundations for further applications of brigatinib and brigatinib-analog.

## 2. Materials and Methods

### 2.1. Reagents and Chemicals

Brigatinib (AP26113, CAS: 1197953-54-0, 99% of purity), brigatinib-analog (AP26113-analog, ALK-IN-1, CAS: 1197958-12-5, 99% of purity), and osimertinib (IS, CAS: 1421373-65-0, 98% of purity) were kindly supplied by the Institute of Materia Medica, Shandong First Medical University & Shandong Academy of Medical Sciences (Jinan, China). HPLC-grade methanol was obtained from Tedia (Fairfield, OH, USA), and acetonitrile, of HPLC grade, was supplied by Fisher Scientific (Fair Lawn, NJ, USA). HPLC-grade formic acid was purchased from Damao Chemical Reagent Factory (Tianjin, China). All other chemicals were of analytic grade or better.

### 2.2. Instruments and LC-MS/MS Conditions

Analyses were acquired on a TSQ Quantum Ultra mass spectrometric detector with electrospray ionization (ESI) interface (Thermo Scientific, USA), which was coupled with a Dionex Ultimate 3000 ultra-performance liquid chromatography system consisting of an LPG-3400SDN pump, a WPS-3000TSL autosampler, and a TCC-3000RS column compartment. Samples were separated on a reversed phase Inertsil ODS-3 column (50 mm × 4.6 mm ID, 5 *μ*m particle size, GL Sciences Inc.) at 25°C and eluted on a gradient mobile phase of water (containing 0.1% formic acid) and acetonitrile at a flow rate of 0.5 mL/min. The consecutive program was as follows: 15% acetonitrile followed by a linear increase to 20% acetonitrile over a 0.2 min period, followed by a linear gradient elution of 20% to 50% acetonitrile from 0.2 to 1.9 min, finally returned to 15% acetonitrile during 0.01 min, and re-equilibrated to its starting conditions until 3 min. The retention times were 2.07, 2.18, and 2.40 min for brigatinib, brigatinib-analog, and the IS, respectively.

**Figure 1:**
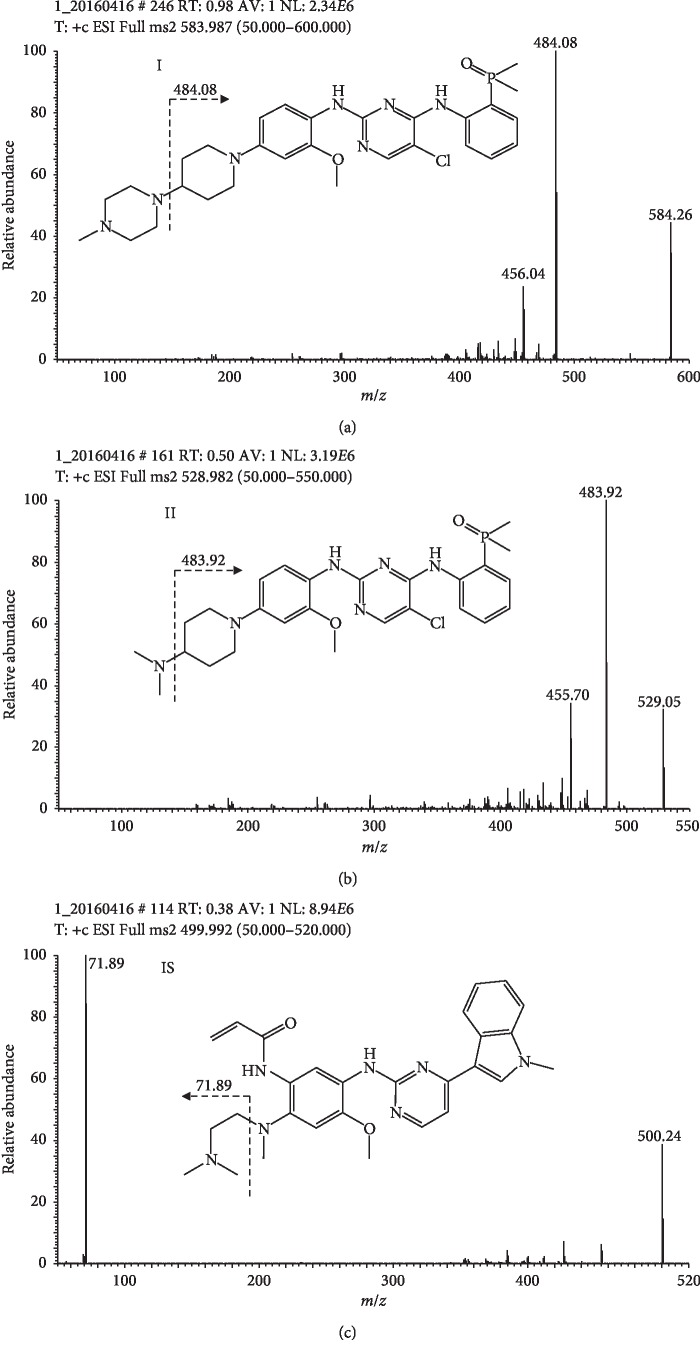
Chemical structures and product ion scan spectra for (a) brigatinib (I); (b) brigatinib-analog (II); (c) IS.

The acquisition was performed using selected reaction monitoring (SRM) of the transitions from protonated precursor ion [M + H]^+^ to the particular daughter ion to quantify each compound. The SRM conditions were defined as follows: spray voltage 3500 V, vaporizer temperature 250°C, sheath gas pressure 35 arb, auxiliary gas pressure 10 arb, and capillary temperature 350°C. The SRM mode of *m/z* 584.26 ⟶ 484.08 [M + H]^+^ for brigatinib, *m/z* 529.05 ⟶ 483.92 [M + H]^+^ for brigatinib-analog, and *m/z* 500.24 ⟶ 71.89 [M + H]^+^ for IS at the positive ionization mode were used as quantitative analysis (Figure 1). All the data were acquired and processed using Xcalibur software (Thermo Scientific, USA).

### 2.3. Preparation of Standard Solutions, Calibration Samples, and Quality Control Samples

Primary stock standard solutions of brigatinib and brigatinib-analog were prepared separately for use as standard and quality controls (QC) at the concentration of 10.0 mg/mL with ethanol and mixed in equal volumes. The mixed standard solution was further serially diluted with methanol. The IS stock standard solution was prepared with dimethyl sulfoxide (DMSO) at the concentration of 1.0 mg/mL and further diluted with methanol to 1000 ng/mL. All standard solutions were kept at 4°C before use.

Calibration standards containing brigatinib and brigatinib-analog were prepared by spiking appropriate amounts of the standard solutions in rat blank plasma or brain homogenate (1 : 99, v/v). Seven levels of the calibration curve were determined (*n* = 5).

The calibration equations were calculated by the least-squares linear regression method. The final concentrations in plasma were 1.0, 4.0, 20, 80, 500, 1000, and 2000 ng/mL for both brigatinib and brigatinib-analog. Quality control (QC) samples were similarly prepared with blank plasma and the final concentrations were 1.0, 2.0, 400, and 1600 ng/mL. The final concentrations in the brain homogenate were 0.5, 2.0, 10, 40, 250, 500, and 1000 ng/mL for both brigatinib and brigatinib-analog. QC samples were both at the concentrations of 0.5, 1.0, 200, and 800 ng/mL. All the spiked samples were treated in accordance with the biosample preparation procedure.

### 2.4. Biosample Preparation

30 *μ*L of rat biosample (plasma or brain homogenate) was mixed with 100 *μ*L of IS solution and 150 *μ*L methanol for protein precipitation. Then, the mixture was vortex-mixed for 1 min and centrifuged at 14,000 rpm for 10 min at 4°C. After centrifugation, the supernatant was carefully separated, and a 10 *μ*L aliquot was injected into the LC-MS/MS system for analysis.

### 2.5. Method Validation

Validation procedures of the developed method were carried out according to US Food and Drug Administration (FDA) guidelines for bioanalytical method validation with respect to selectivity, sensitivity, linearity, intrabatch and interbatch precision and accuracy, recovery, matrix effect, stability, and carryover [16].

### 2.6. Application to Pharmacokinetic and Brain Distribution Study

Seventy-two male Sprague Dawley rats weighing 180–220 g (7-8 weeks old, Certificate no. SCXK (Shandong) 2014-0007) were supplied by Shandong Laboratory Animal Center, Shandong First Medical University & Shandong Academy of Medical Sciences. All rats were kept in plastic cages with 12 h day/night cycle under controlled temperature (about 23–25°C) and fed with standard laboratory rat diet and water *ad libitum*. All the animal welfare and experimental procedures were approved by the Animal Ethics Committee at the Institute of Materia Medica, Shandong First Medical University & Shandong Academy of Medical Sciences (Jinan, China) and strictly in accordance with the guide for the care and use of laboratory animals (National Research Council of USA, 1996).

For the pharmacokinetic studies, after fasting with free access to water for at least 12 h, twelve rats were randomly divided into two groups (*n* = 6). The brigatinib and brigatinib-analog were both dissolved in ethanol-0.5% methylcellulose (1 : 19) for dosing. The two group rats were intragastrically administered with brigatinib and brigatinib-analog at the same dose of 5 mg/kg, respectively.

Blood samples, about 100 *μ*L of each, were placed in heparinized Eppendorf tubes predose and at 5, 15, 30, 60, 120, 180, 240, 300, 360, 480, 720, and 1,440 min postdose from the jugular vein of rats. The blood samples were immediately centrifuged at 3,500 rpm for 15 min at 4°C, and the plasma samples were transferred carefully from each collection and stored at −20°C until analysis [17, 18].

For the brain distribution studies, the remaining sixty rats were randomly divided into ten groups (*n* = 6, per group). The distributions of brigatinib and brigatinib-analog in the rat brain were also investigated at the dose of 5 mg/kg after single oral administration to rats. The whole brain was excised immediately at 5, 60, 240, 480, and 1,440 min postdose, thoroughly rinsed with 0.9% saline solution, and wiped with a filter paper. Each brain sample was weighed accurately and homogenized in double distilled water (1 : 5, w/v) by using a tissue homogenizer (IKA, Germany) and frozen at −20°C until analysis [19].

### 2.7. Data Analysis

Experimental data were expressed as mean ± standard deviation (SD). Drug and Statistics 2.0 (DAS 2.0) software package (Mathematical Pharmacology Professional Committee of China, Shanghai, China) was used for estimating pharmacokinetic and brain distribution parameters by a noncompartmental method. Statistical analyses were performed with statistical software IBM SPSS Statistics 20.0 (IBM Corporation, Armonk, NY, USA). Differences between two groups were evaluated by the *t*-test. A value of *p* < 0.05 was considered as statistically significant.

## 3. Results and Discussion

### 3.1. Optimization of LC-MS Conditions

#### 3.1.1. Mass Spectra

The analytes and IS were found to respond best to positive ionization with the adduct ions [M + H]^+^, which were presented as the major peaks. Their product ion mass spectra are shown in Figure 1. The SRM mode was used to monitor the transitions of *m/z* 584.26 ⟶ 484.08 for brigatinib, *m/z* 529.05 ⟶ 483.92 for brigatinib-analog, and *m/z* 500.24 ⟶ 71.89 for IS.

#### 3.1.2. Liquid Chromatography

When selecting the mobile phase for the LC-MS/MS system, attention was paid to the influence of the mobile phase on the chromatographic retention and the MS response. Formic acid modifier can improve the ionization efficiency. Different concentrations of formic acid at levels of 0.05%, 0.1%, and 0.2% were examined in the aqueous portion of the mobile phase. The results indicated that adding 0.1% formic acid in the aqueous portion was sufficient to achieve the highest MS sensitivity for brigatinib, brigatinib-analog, and IS. After lots of preliminary experiments had been done, it was found that an Inertsil ODS-3 column (50 mm × 4.6 mm ID, 5 *μ*m particle size) produced the best separation of the two compounds. The mobile phase consisted of water (containing 0.1% formic acid) and acetonitrile with gradient elution, which was used for narrowing the peaks and improving the resolution of analytes and IS and shorting the running time of the chromatography.

In addition, owing to the complex nature of rat plasma and brain homogenate, the pretreatment of biosample is a significant step to remove protein and potential interferences prior to LC-MS/MS analysis. The extraction conditions, including protein precipitation, liquid-liquid extraction, and solid-phase extraction were investigated. The specificity, sensitivity, stability, and accuracy of protein precipitation, which required less time and decreased the cost of the assay, could meet the requirements of the research.

### 3.2. Method Validation

#### 3.2.1. Selectivity

**Figure 2:**
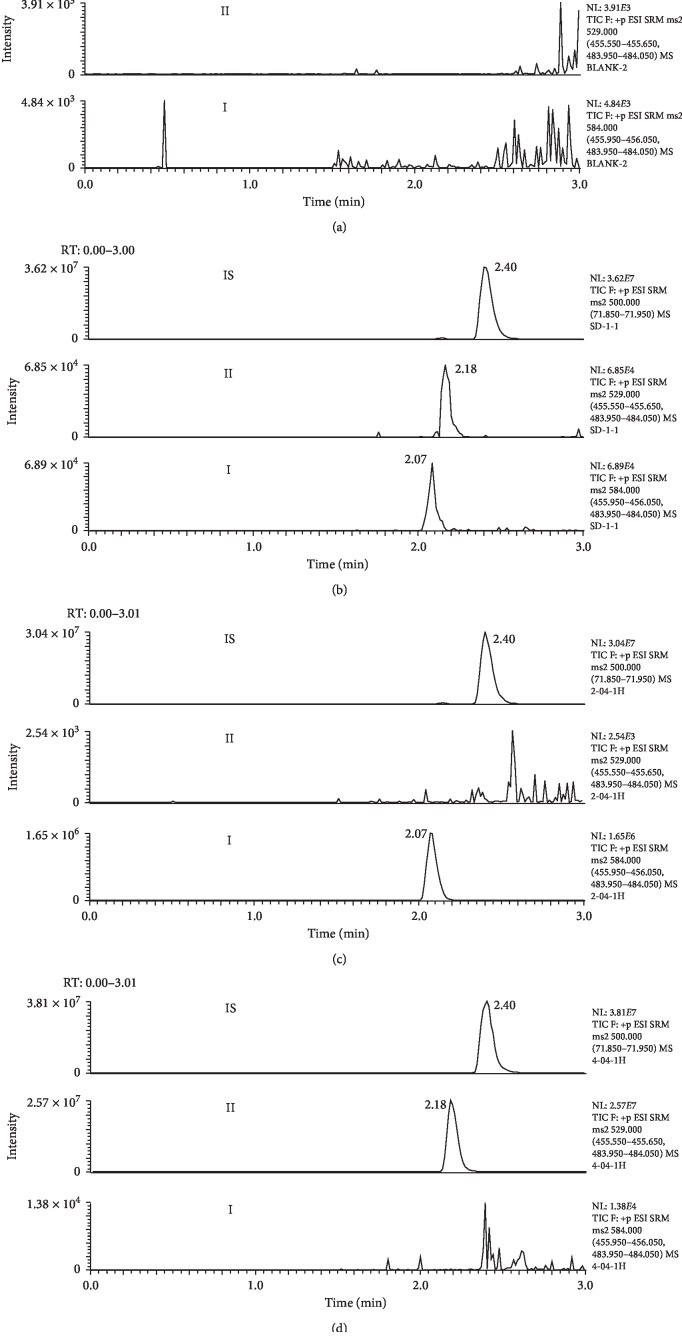
SRM chromatograms for brigatinib (I), brigatinib-analog (II), and IS in rat plasma: (a) blank rat plasma; (b) blank plasma spiked with the analytes (1.0 ng/mL) and IS; (c) a rat plasma sample collected 1 h after oral administration of 5.0 mg/kg brigatinib; (d) a rat plasma sample collected 1 h after oral administration of 5.0 mg/kg brigatinib-analog.

Six different batches of blank biosample obtained from six different sources were prepared. No interferences from endogenous substances of plasma and brain homogenate were observed in the retention regions of either analytes or IS (Figure 2 and Supplementary materials [Supplementary-material supplementary-material-1]).

#### 3.2.2. Linearity and LLOQ

The calibration curves were obtained by plotting the peak area ratio of the analytes to IS against the corresponding concentration of the analytes in the freshly prepared rat biomatrix calibrators. The linear regression equations for brigatinib and brigatinib-analog in rat plasma were *y* = 5.65 × 10^−4^*x* + 2.81 × 10^−4^ and *y* = 5.21 × 10^−4^*x* + 5.78 × 10^−4^, respectively. The mean correlation coefficients (*r*^2^) of calibration curves were all >0.99 over the concentration range from 1.0 to 2000 ng/mL with the weighting factor of 1/*x*^2^. The linear regression equations for brigatinib and brigatinib-analog in the rat brain homogenate over the concentration range from 0.5 to 1000 ng/mL with the weighting factor of 1/*x*^2^ were *y* = 7.43 × 10^−4^*x* − 2.23 × 10^−4^ (*r*^2^ = 0.9904) and *y* = 6.51 × 10^−4^*x* + 2.33 × 10^−4^ (*r*^2^ = 0.9919), respectively. The lower limits of quantification (LLOQs) for brigatinib and brigatinib-analog were both found to be 1.0 ng/mL in rat plasma and they were 0.5 ng/mL in the rat brain homogenate.

#### 3.2.3. Precision and Accuracy

The four different QC levels were used to assess the intrabatch and interbatch precision and accuracy of brigatinib and brigatinib-analog in rat plasma and brain homogenate by analyzing six replicates of each QC level on three separate batches using independently prepared calibration curves. The assay values were less than 15% for all concentrations (<20% for LLOQ) and conformed to the accepted variable limits, which demonstrated that the method was reliable and reproducible for quantification of brigatinib and brigatinib-analog in rat plasma and brain homogenate (Table 1).

#### 3.2.4. Extraction Recovery and Matrix Effect

The responses of analytes from low, medium, and high QC samples with known amounts (A), analytes dissolved in the postextracted blank matrix at the same QC concentration levels (B), and neat standards at the same QC concentration levels (C) were measured, respectively (*n* = 3). The ratio of A against C was used to estimate the extraction recovery, while the ratio of B against C was used to evaluate the matrix effect (Table 2). The results suggested that the extraction recovery was within the acceptable range, and there was no significant matrix effect on brigatinib and brigatinib-analog in this method.

#### 3.2.5. Stability and Carryover

The stability of brigatinib and brigatinib-analog was evaluated by analysis of low and high concentration levels of QC samples under different conditions (*n* = 6). The temperature and timing conditions were as follows: 6 h at ambient temperature, stored at −20°C for 10 d, three freeze-thaw cycles, and 24 h at 4°C in an autosampler. The measured concentrations were all within acceptable limits (±15% of the nominal concentrations) during the entire validation. The results indicated that brigatinib and brigatinib-analog were stable under conditions investigated in this study (Table 3). Carryover was evaluated by determining the peak areas for brigatinib and brigatinib-analog by injecting blank matrix samples sequentially after the highest calibration standard sample injection. Furthermore, no carryover effect was obtained in this study.

### 3.3. Pharmacokinetic and Brain Distribution Application

The validated method was successfully applied to study the pharmacokinetics of brigatinib and brigatinib-analog in rats by gavage at the dose of 5 mg/kg, respectively.

**Figure 3:**
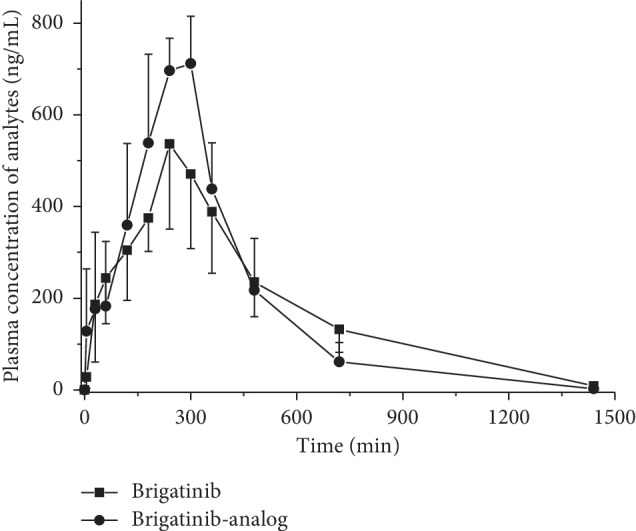
Plasma concentration-time profiles of brigatinib and brigatinib-analog following single oral administration of 5 mg/kg of brigatinib and brigatinib-analog in male SD rats (*n* = 6).

No significant differences in area under the curve (AUC; 261528.73 ± 86227.28 vs. 262436.45 ± 74089.38 ng/mL·min), peak time (*T*_max_; 260.00 ± 30.98 vs. 260.00 ± 48.99 min), maximum concentration (*C*_max_; 537.85 ± 185.55 vs. 734.41 ± 83.06 ng/mL), apparent volume of distribution (*V*_*d*_; 5.66 ± 1.94 vs. 3.43 ± 0.96 L/kg), and clearance (*CL*_*z*_*/F*; 0.022 ± 0.010 vs. 0.021 ± 0.006 L/min/kg) were observed between brigatinib and brigatinib-analog in rats. The mean residence time (MRT) and half-life (*T*_1*/*2_) of brigatinib in rats were 403.98 ± 12.02 min and 189.41 ± 20.55 min. Meanwhile, the same parameters of brigatinib-analog exhibited significant decreases in MRT (328.55 ± 35.68 min) and *T*_1*/*2_ (120.78 ± 29.74 min) compared with those of brigatinib (*p* < 0.05). The mean plasma concentration-time profiles of brigatinib and brigatinib-analog in rats are summarized in Figure 3.

**Figure 4:**
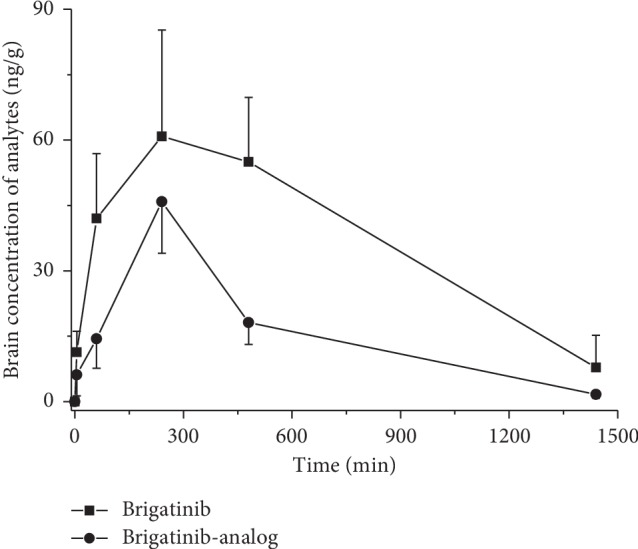
Brain concentration-time profiles of brigatinib and brigatinib-analog following single oral administration of 5 mg/kg of brigatinib and brigatinib-analog in male SD rats (*n* = 6).

The established LC-MS/MS method was also successfully applied to investigate the brain distribution of brigatinib and brigatinib-analog in rats following oral administration of 5 mg/kg. The brain distribution researches found that the brigatinib and brigatinib-analog could both penetrate the blood-brain barrier (BBB). The ability of analytes penetrating the BBB was estimated by using the ratio of average AUC_(0*–t*)_ in the rat brain to that in the plasma (AUC_brain_/AUC_plasma_) at the same dose, supposing 1 g of brain tissue was equivalent to 1 mL of plasma. The ratio of AUC_brain_/AUC_plasma_ was 0.223 and 0.088 for brigatinib and brigatinib-analog, respectively, which meant that brigatinib has stronger ability passing through the BBB than brigatinib-analog in rats. In addition, there were significant differences in AUC_(*0–t*)_, MRT, *C*_max_, *V*_*d*_, and *CL*_*z*_*/F* between brigatinib and brigatinib-analog in the rat brain (*p* < 0.05). The mean brain tissue concentration-time profiles of brigatinib and brigatinib-analog following single oral administration in rats are presented in Figure 4. The main pharmacokinetic parameters of brigatinib and brigatinib-analog in rat plasma and brain tissue are presented in Table 4.

## 4. Conclusion

In conclusion, a highly sensitive, reliable, and simple LC-MS/MS method had been developed and validated for the simultaneous determination of brigatinib and brigatinib-analog in rat plasma and brain homogenate. The validated LC-MS/MS method was also successfully applied to the pharmacokinetics and brain distribution studies of brigatinib and brigatinib-analog in rats after a single oral administration of 5 mg/kg. The pharmacokinetic researches suggested that there remained some differences in pharmacokinetic characteristics between brigatinib and brigatinib-analog in rats. Moreover, compared with brigatinib-analog, brigatinib was a stronger central nervous system-penetrant. However, the future study should increase sample size to make the empirical conclusions more convincing. The above studies would lay a good foundation for the further applications of brigatinib and brigatinib-analog.

## Figures and Tables

**Table 1:** The intrabatch and interbatch precision and accuracy of brigatinib and brigatinib-analog in rat plasma and brain homogenate (*n* = 3 batches, 6 replicates per batch).

Analytes	Matrix	Spiked conc. (ng/mL)	Intrabatch (*n* = 6)			Interbatch (*n* = 18)		
			Measured conc. (ng/mL)	Precision (CV%)	Accuracy (%)	Measured conc. (ng/mL)	Precision (CV%)	Accuracy (%)
Brigatinib	Plasma	1.0	0.92 ± 0.11	11.95	92.00	0.95 ± 0.10	10.52	95.00
		2.0	2.03 ± 0.21	10.34	101.50	1.97 ± 0.18	9.14	98.50
		400	391.88 ± 28.99	7.39	97.97	408.28 ± 38.34	9.39	102.07
		1600	1514.40 ± 96.16	6.35	94.65	1548.96 ± 67.38	4.35	96.81
	Brain homogenate	0.5	0.45 ± 0.07	15.56	90.00	0.52 ± 0.06	11.53	104.00
		1.0	1.05 ± 0.11	10.47	105.00	0.96 ± 0.08	8.33	96.00
		200	190.78 ± 19.48	10.21	95.39	192.07 ± 14.10	7.34	95.39
		800	845.20 ±71.13	8.42	105.65	813.20 ± 51.13	6.29	101.65
Brigatinib-analog	Plasma	1.0	1.07 ± 0.13	12.15	107.00	1.06 ± 0.11	10.38	106.00
		2.0	1.87 ± 0.15	8.02	93.50	1.92 ± 0.14	7.29	96.00
		400	422.46 ± 39.09	9.25	105.62	402.53 ± 29.17	7.25	100.63
		1600	1537.96 ± 101.46	6.60	96.12	1557.67 ± 86.35	5.54	97.35
	Brain homogenate	0.5	0.53 ± 0.09	16.98	106.00	0.51 ± 0.07	13.73	102.00
		1.0	0.96 ± 0.10	10.42	96.00	0.97 ± 0.08	8.25	97.00
		200	208.54 ± 16.83	8.07	104.27	210.19 ± 15.26	7.26	105.10
		800	764.06 ± 54.21	7.09	95.51	781.64 ± 47.34	6.06	97.71

**Table 2:** Extraction recovery and matrix effect of brigatinib and brigatinib-analog in rat plasma and brain homogenate (mean ± SD, *n* = 3).

Analytes	Matrix	Spiked conc. (ng/mL)	Extraction recovery (%)		Matrix effect (%)	
			Mean ± SD	RSD	Mean ± SD	RSD
Brigatinib	Plasma	2.0	102.37 ± 9.23	9.02	92.80 ± 11.08	11.94
		400	97.61 ± 5.55	5.69	98.62 ± 7.10	7.19
		1600	93.10 ± 4.46	4.79	102.97 ± 8.82	8.57
	Brain homogenate	1.0	88.28 ± 5.13	5.81	93.78 ± 9.43	10.06
		200	86.81 ± 4.83	5.56	95.56 ± 8.47	8.86
		800	89.74 ± 4.50	5.01	96.62 ± 8.12	8.40
Brigatinib-analog	Plasma	2.0	112.68 ± 10.89	9.66	97.89 ± 12.09	12.35
		400	106.42 ± 5.32	4.99	95.99 ± 6.41	6.68
		1600	108.24 ± 7.72	7.13	95.94 ± 8.58	8.94
	Brain homogenate	1.0	97.43 ± 7.03	7.22	97.68 ± 7.50	7.68
		200	91.66 ± 5.43	5.92	102.14 ± 3.52	3.45
		800	98.65 ± 8.66	8.78	94.06 ± 6.15	6.54

**Table 3:** Stability data of brigatinib and brigatinib-analog in rat plasma and brain homogenate under various storage conditions at two QC levels (*n* = 6).

Analytes	Matrix	Spiked conc. (ng/mL)	Storage conditions (ng/mL)			
			Ambient temperature for 6 h	−20°C for 10 d	Three freeze-thaw cycles	Autosampler at 4°C for 24 h
Brigatinib	Plasma	2.0	2.10 ± 0.12	2.05 ± 0.17	1.94 ± 0.09	1.90 ± 0.10
		1600	1585.06 ± 98.31	1719.73 ± 107.09	1678.34 ± 95.87	1645.17 ± 118.52
	Brain homogenate	1.0	1.06 ± 0.08	0.97 ± 0.06	1.02 ± 0.06	0.96 ± 0.05
		800	788.85 ± 69.25	760.29 ± 48.33	847.44 ± 57.65	813.16 ± 72.23
Brigatinib-analog	Plasma	2.0	2.03 ± 0.11	1.95 ± 0.12	2.01 ± 0.09	1.89 ± 0.12
		1600	1638.54 ± 97.76	1705.49 ± 109.83	1578.36 ± 125.20	1582.61 ± 101.88
	Brain homogenate	1.0	1.04 ± 0.05	1.07 ± 0.11	0.98 ± 0.07	0.97 ± 0.06
		800	828.64 ± 66.54	846.09 ± 53.83	833.68 ± 56.59	783.28 ± 32.39

**Table 4:** Pharmacokinetic parameters of brigatinib and brigatinib-analog after oral administration of 5 mg/kg of brigatinib and brigatinib-analog in male SD rats (mean ± SD, *n* = 6).

Analytes	Units	Plasma		Brain	
		Brigatinib	Brigatinib-analog	Brigatinib	Brigatinib-analog
AUC_(*0–t*)_	ng/mL·min (plasma), ng/g·min (brain)	261528.73 ± 86227.28	262436.45 ± 74089.38	58255.81 ± 13725.10	23196.08 ± 4932.95^*∗*^
MRT	min	403.98 ± 12.02	328.55 ± 35.68^*∗*^	449.04 ± 65.44	379.04 ± 5.40^*∗*^
*T*_1*/*2_	min	189.41 ± 20.55	120.78 ± 29.74^*∗*^	319.42 ± 117.05	256.08 ± 40.01
*T*_max_	min	260.00 ± 30.98	260.00 ± 48.99	330.00 ± 176.98	240.00 ± 0.00
*C*_max_	ng/mL (plasma), ng/g (brain)	537.85 ± 185.55	734.41 ± 83.06	68.91 ± 21.57	45.84 ± 11.81^*∗*^
*V*_*d*_	L/kg	5.66 ± 1.94	3.43 ± 0.96	50.47 ± 19.42	119.35 ± 25.58^*∗*^
*CL*	L/min/kg	0.022 ± 0.010	0.021 ± 0.006	0.101 ± 0.062	0.325 ± 0.057^*∗*^

^*∗*^ *p* < 0.05 compared with brigatinib.

## Data Availability

The datasets generated and analysed during the current study are not publicly available due to confidentiality agreement of the institution but are available from the corresponding author on reasonable request.

## Supplementary Materials

Supplementary Materials Supplementary Figure 1: SRM chromatograms for brigatinib (I) and brigatinib-analog (II) and IS in the rat brain homogenate: (A) blank rat brain homogenate; (B) blank brain homogenate spiked with the analytes (0.5 ng/mL) and IS; (C) a rat brain homogenate sample collected 4 h after single oral administration of 5.0 mg/kg brigatinib; (D) a rat brain homogenate sample collected 4 h after single oral administration of 5.0 mg/kg brigatinib-analog.

## References

[1] Chan B. A., Hughes B. G. (2015). Targeted therapy for non-small cell lung cancer: current standards and the promise of the future. *Translational Lung Cancer Research*.

[2] Chan B. A., Coward J. I. (2013). Chemotherapy advances in small-cell lung cancer. *Journal of Thoracic Disease*.

[3] Rossi A., Maione P., Sacco P. C. (2014). ALK inhibitors and advanced non-small cell lung cancer (review). *International Journal of Oncology*.

[4] Huang W.-S., Liu S., Zou D. (2016). Discovery of brigatinib (AP26113), a phosphine oxide-containing, potent, orally active inhibitor of anaplastic lymphoma kinase. *Journal of Medicinal Chemistry*.

[5] Beardslee T., Lawson J. (2018). Alectinib and brigatinib: new second-generation ALK inhibitors for the treatment of non-small cell lung cancer. *Journal of the Advanced Practitioner in Oncology*.

[6] Fontana D., Ceccon M., Gambacorti-Passerini C., Mologni L. (2015). Activity of second-generation ALK inhibitors against crizotinib-resistant mutants in an NPM-ALK model compared to EML4-ALK. *Cancer Medicine*.

[7] Roskoski R. (2017). Anaplastic lymphoma kinase (ALK) inhibitors in the treatment of ALK-driven lung cancers. *Pharmacological Research*.

[8] Hamedani F. S., Cinar M., Mo Z., Cervania M.A., Amin H.M., Alkan S. (2014). Crizotinib (PF-2341066) induces apoptosis due to downregulation of pSTAT3 and BCL-2 family proteins in NPM-ALK(+) anaplastic large cell lymphoma. *Leukemia Research*.

[9] Markham A. (2017). Brigatinib: first global approval. *Drugs*.

[10] Mezquita L., Planchard D. (2018). The role of brigatinib in crizotinib-resistant non-small cell lung cancer. *Cancer Management and Research*.

[11] Iwama E., Okamoto I., Harada T., Takayama K., Nakanishi Y. (2014). Development of anaplastic lymphoma kinase (ALK) inhibitors and molecular diagnosis in ALK rearrangement-positive lung cancer. *OncoTargets and Therapy*.

[12] Uchibori K., Inase N., Araki M. (2017). Brigatinib combined with anti-EGFR antibody overcomes osimertinib resistance in EGFR-mutated non-small-cell lung cancer. *Nature Communications*.

[13] Zhang S., Anjum R., Squillace R. (2016). The potent ALK inhibitor brigatinib (AP26113) overcomes mechanisms of resistance to first- and second-generation ALK inhibitors in preclinical models. *Clinical Cancer Research*.

[14] Wen J., Bao S., Cai Y. (2017). A reliable and stable method for determination of brigatinib in rat plasma by UPLC-MS/MS: application to a pharmacokinetic study. *Journal of Chromatography B*.

[15] Sparidans R. W., Li W., Schinkel A. H., Schellens J. H. M., Beijnen J. H. (2018). Bioanalytical liquid chromatography-tandem mass spectrometric assay for the quantification of the ALK inhibitors alectinib, brigatinib and lorlatinib in plasma and mouse tissue homogenates. *Journal of Pharmaceutical and Biomedical Analysis*.

[16] US-FDA (2018). *Bioanalytical Method Validation Guidance for Industry*.

[17] Cui W. J., Liu Q., Xiong S., Qiao L. (2018). LC-MS/MS method for Simultaneous determination of indapamide, perindopril and perindoprilat in human plasma or whole blood by UPLC-MS/MS and its pharmacokinetic application. *Journal of Analytical Methods in Chemistry*.

[18] Jiang L., Xiong Y. L., Yu L. B. (2019). Development of a UPLC-MS/MS method for determination of pimavanserin tartrate in rat plasma: application to a pharmacokinetic study. *International Journal of Analytical Chemistry*.

[19] Xiong S., Xue M., Mu Y., Deng Z., Sun P., Zhou R. (2017). Determination of AZD3759 in rat plasma and brain tissue by LC-MS/MS and its application in pharmacokinetic and brain distribution studies. *Journal of Pharmaceutical and Biomedical Analysis*.

