# Supplementary material for: Simultaneous Quantification of Brigatinib and Brigatinib-Analog in Rat Plasma and Brain Homogenate by LC-MS/MS: Application to Comparative Pharmacokinetic and Brain Distribution Studies
Source: Int J Anal Chem. 2019 Dec 5;2019:9028309. doi: 10.1155/2019/9028309 (PMC6915135; doi:10.1155/2019/9028309)

**Supplementary materials**

**Supplementary Figure 1.** SRM chromatograms for brigatinib (I) and brigatinib-analog (II) and IS in rat brain homogenate.

(A) blank rat brain homogenate; (B) blank brain homogenate spiked with the analytes (0.5 ng/mL) and IS; (C) a rat brain homogenate sample collected 4 h after single oral administration of 5.0 mg/kg brigatinib; (D) a rat brain homogenate sample collected 4 h after single oral administration of 5.0 mg/kg brigatinib-analog.


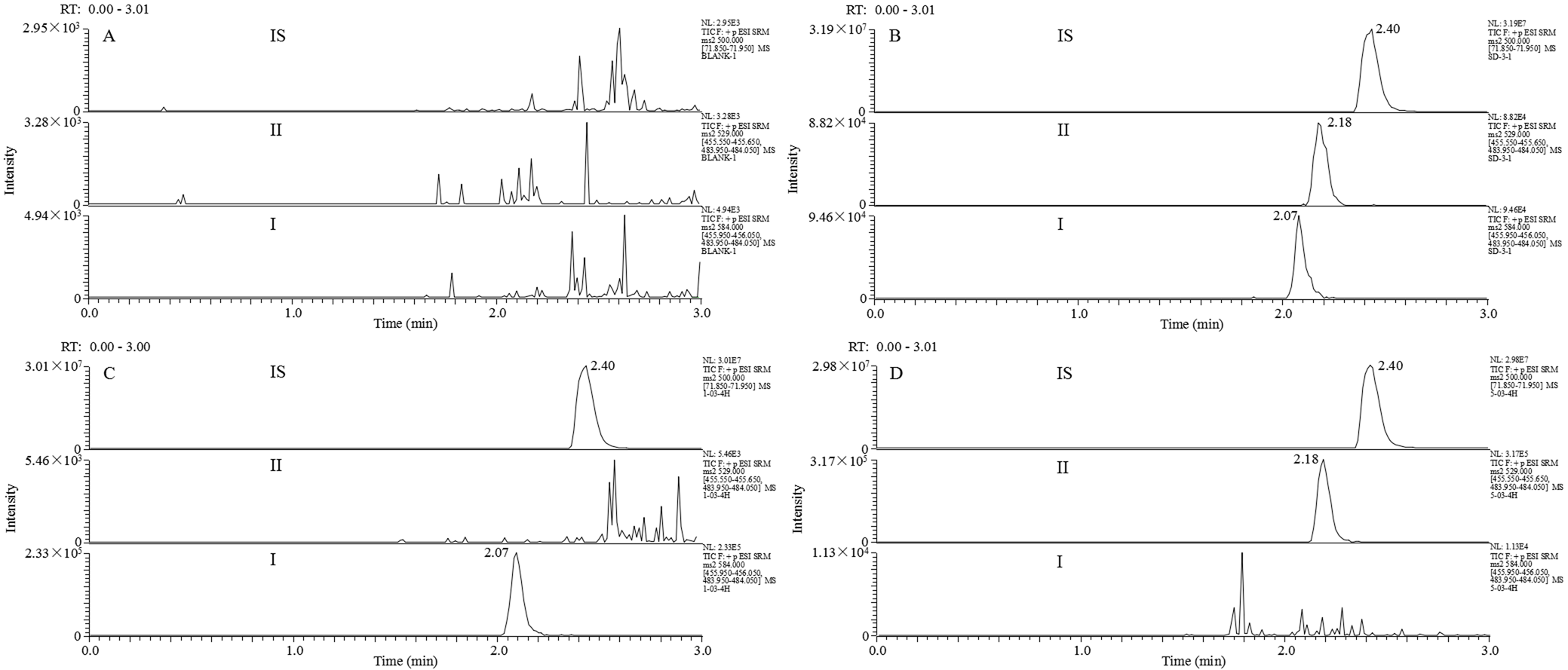

Supplement: Supplementary Materials — Supplementary Figure 1: SRM chromatograms for brigatinib (I) and brigatinib-analog (II) and IS in the rat brain homogenate: (A) blank rat brain homogenate; (B) blank brain homogenate spiked with the analytes (0.5 ng/mL) and IS; (C) a rat brain homogenate sample collected 4 h after single oral administration of 5.0 mg/kg brigatinib; (D) a rat brain homogenate sample collected 4 h after single oral administration of 5.0 mg/kg brigatinib-analog. [file 9028309.f1.doc]
